# A novel nomogram and prognostic factor for metastatic soft tissue sarcoma survival

**DOI:** 10.3389/fendo.2024.1371910

**Published:** 2024-05-13

**Authors:** Dan Han, Bing Li, Jie Xu, Yajie Hu, Xi Chen, Ruizhi Wang

**Affiliations:** ^1^ Department of Pharmacy, Huadong Hospital, Fudan University, Shanghai, China; ^2^ Department of Radiology, Seventh People's Hospital of Shanghai University of Traditional Chinese Medicine (TCM), Shanghai, China; ^3^ Department of Radiology, Huadong Hospital, Fudan University, Shanghai, China; ^4^ Department of Oncology, Huadong Hospital, Fudan University, Shanghai, China

**Keywords:** metastatic soft tissue sarcoma, immune checkpoint inhibitors, nomogram, survival, SEER database

## Abstract

**Background:**

This study represented the inaugural effort to develop predictive survival nomograms for metastatic soft tissue sarcoma (mSTS) patients in the era of immune checkpoint inhibitors.

**Method:**

From the Surveillance, Epidemiology, and End Results (SEER) program database, we extracted 3078 eligible patients with mSTS between 2016 and 2022. Kaplan-Meier survival analysis, univariate and multivariable Cox analyses, and univariate and multivariable logistic analyses were conducted. Subsequently, predictive nomograms were constructed. Clinical effectiveness was validated using the area under the curve (AUC), calibration curve, and decision curve analysis (DCA) methods.

**Results:**

We used the SEER database to include 3078 eligible patients with mSTS between 2016 and 2022. All the eligible patients were randomly allocated in a ratio of 6:4 and stratified into a training group (n = 1846) and a validation group (n = 1232). In the multivariate Cox analysis, age, race, marital status, pathological grade, histologic subtype, surgery, and chemotherapy were identified as independent prognostic factors. These factors were used to construct the nomogram to predict the 1-, 3-, and 5-year OS of mSTS patients. The C-index for the training cohort and the validation cohort was 0.722(95% confidence interval [CI]: 0.708–0.736), and 0.716(95% CI: 0.698–0.734), respectively. The calibration curves for 1-, 3-, and 5-year OS probability demonstrated excellent calibration between the predicted and the actual survival. The AUC values of the nomogram at 1-, 3-, and 5-year were 0.785, 0.767, and 0.757 in the training cohort, 0.773, 0.754, and 0.751 in the validation cohort, respectively. Furthermore, DCA indicated the favorable clinical utility of the nomogram in both cohorts. The risk stratification system was constructed using the established nomogram, which enhanced prediction accuracy, aided clinicians in identifying high-risk patients and informing treatment decisions.

**Conclusion:**

This study marked the inaugural effort in constructing predictive survival nomograms mSTS patients in the era of immune checkpoint inhibitors. The robustly constructed nomograms, alongside actual outcomes, offered valuable insights to inform follow-up management strategies.

## 1 Introduction

Soft tissue sarcoma (STS) is a highly heterogeneous malignant tumor originating from mesenchymal tissues, accounting for approximately 0.72% to 1.05% of human malignancies (1). However, STS exhibits complex histological subtypes (over 70), and significant differences in biological characteristics and treatment responses exist among these subtypes, posing challenges to clinical research in the field and contributing to a relatively slow overall therapeutic progress (2). STS displays a relatively low sensitivity to radiation and chemotherapy. Surgical intervention remains the primary treatment for early-stage STS, with a 5-year overall survival rate ranging from 60% to 80% (2). Nevertheless, more than half of the patients may experience local recurrence or distant metastasis after surgery, and the average survival period for patients with distant metastasis is only 12 to 18 months (3). STS primarily metastasizes to the lungs, with isolated pulmonary metastasis observed in as many as 52% of metastatic cases (3). In addition, metastases can occur in other locations such as the liver, bones, and brain (4).

Treatment options for metastatic STS (mSTS) are limited, with first-line standard regimens typically based on chemotherapy, specifically anthracycline-based chemotherapy, but with a modest efficacy of only 10% to 25% (4). Other chemotherapy drugs, such as ifosfamide (5), docetaxel/gemcitabine (6), trabectedin (7), eribulin (8), demonstrate similar or lower efficacy rates. Currently, molecular targeted drugs, including anlotinib (9) and pazopanib (10), have been approved for second-line treatment of advanced STS, yet the median progression-free survival for patients remains merely 5.6 months. Immunotherapy, as a distinct approach from traditional treatments, relies on stimulating the host’s immune system to naturally attack malignant tumor cells. Immunotherapy has demonstrated positive outcomes in various cancers and has even altered the treatment landscape and outcomes of refractory tumors such as malignant melanoma. Since 2011, the FDA has sanctioned numerous immunotherapeutic drugs for cancer, signifying a substantial proliferation of treatment avenues for individuals with metastatic STS within the past decade. Targeted immune checkpoint inhibitors (ICIs) have emerged as a novel and pivotal modality in treatment. The ICIs have transformed the therapy landscape for mSTS (11). The utilization of ICIs in the management of mSTS, whether as standalone treatments or in conjunction with other protocols, has progressed in recent years, yielding promising outcomes. Presently, there have been several randomized trials exploring ICIs, either individually or in combination with other ICIs and VEGF/VEGF-R targeted drugs. These include nivolumab in combination with or without ipilimumab in the Alliance A091401 clinical study (12), nivolumab in combination with sunitinib in the MMUNOSARC trials (13). These trials collectively suggest a viable therapeutic option for individuals with mSTS.

While these drugs are commonly employed for the management of mSTS, there has been a dearth of population-based investigations examining their survival benefits in individuals with metastasis disease in the era of ICIs.

Furthermore, the rarity of STS presents a considerable obstacle when attempting to conduct randomized controlled trials for a comprehensive and systematic comparison of various treatment outcomes. Consequently, our understanding of STS is often derived from retrospective studies, given the limited feasibility of prospective trials in this context. While the prevailing emphasis in existing retrospective studies predominantly centers on evaluating the prognostic implications associated with surgical interventions (14, 15), there exists a conspicuous gap in the exploration of factors that impact the effectiveness of chemotherapy and radiotherapy in the era of ICIs (11). This identified void in the literature underscores the pressing need for additional and targeted investigative endeavors in this specific domain of research. Addressing these gaps will not only contribute to a more holistic comprehension of STS but also offer valuable insights into optimizing treatment strategies in the era of ICIs (16). In light of the challenges posed by the rarity of STS, collaborative efforts in the form of multi-institutional studies and data pooling may prove instrumental in amassing a sufficiently large cohort for more robust analyses. Hence, researches focus on the effectiveness of chemotherapy and radiotherapy in STS are essential for refining treatment approaches in this unique and challenging context.

Nomograms have emerged as reliable predictive tools, amalgamating diverse clinicopathological data to forecast prognosis (17–19). The Sarculator retroperitoneal sarcoma (RPS) system is often used to guide treatment plans for STS patients (20, 21). However, the prognosis of these patients can be influenced by factors such as race, socioeconomic status, and sex. Relying solely on this system to predict prognoses for all patients may lead to inaccuracies due to its limitations. Ignoring key variables can lead to varying survival rates even among patients with similar disease stages. Thus, new prognostic approaches are needed to enhance the accuracy of survival predictions for SCM patients given the clinical singularity of STS. furthermore, due to the rarity and histological heterogeneity of STS, most analyses of prognostic factors have been derived from retrospective, single-center studies with limited sample sizes (22–25). As far as our knowledge extends, no nomogram has been developed using mSTS patient data from a national database in the era of ICIs. The Surveillance, Epidemiology, and End Results (SEER) database, encompassing demographics, socioeconomic status, and survival data from diverse cancer patients in population-based cancer registries, offers valuable resources for investigating uncommon tumors (26). This study utilized the SEER-18 database to systematically evaluate the overall survival(OS) rates among patients with metastatic STS during the era of ICIs. In addition, Thus, we aimed to construct and validate the initial comprehensive and pragmatic mSTS nomograms, leveraging a large sample size from the SEER database to enhance prognostic predictions for OS in the era of ICIs.

## 2 Patients and methods

### 2.1 Data source and study design

The data utilized in this study were sourced from the SEER database. SEER, a population-based cancer dataset, archives a comprehensive array of patient and tumor-related information, encompassing incidence, survival, mortality, and additional characteristic. Using the SEER*Stat software, we obtained data by specifically selecting cases histologically diagnosed with STS based on the 5th edition of the WHO Classification of Tumors (27), as the first and only primary tumor within the years 2016–2022. According to FDA guidelines, these cases were treated with ICIs, and only adult patients with stage IV cancer were included according to the 8th edition of the American Joint Committee on Cancer (AJCC) TNM staging criteria. This data period corresponds to the era of ICIs treatment. Exclusion criteria for the study comprised were used based on a recent study, which focuses on STS (28).

### 2.2 Variables and outcomes

The following variables that could potentially influence survival were chosen for analysis, including age, race, gender, marital status, pathological grade, tumor size, TNM stage, distant metastatic site, histologic subtype, chemotherapy, radiotherapy, surgery and survival outcomes (time and status). The main endpoints encompassed overall survival (OS), which was determined as the complete duration of survival from the diagnosis of STS to death caused by STS or other factors. The baseline clinical characteristics of patients underwent a two-tailed test using the chi-square method, and a significance level of P < 0.05 was deemed statistically meaningful.

### 2.3 Development and validation of the predictive nomogram

Eligible cases were randomly assigned to the training and internal validation cohorts in a 6:4 ratio. The training cohort was utilized for the construction of nomogram and the development of the risk stratification system. Meanwhile, the validation cohort was employed to assess both the nomogram and risk stratification system. The univariate Cox analysis was employed to identify statistically significant variables for inclusion in the multivariate Cox analysis. Subsequently, the nomogram was constructed based on the independent prognostic factors derived from the multivariate Cox analysis. Nomogram validation involved assessing calibration ability through calibration curves, estimating discrimination ability using the concordance index (C-index), and receiver operating characteristic (ROC) curve. Clinical utility was investigated through the decision curve analysis (DCA), enabling researchers to evaluate the nomogram’s practical utility in guiding clinical decisions (29). Survival analysis was conducted utilizing the Kaplan-Meier methodology, with the log-rank test employed to investigate survival disparities.

## 3 Results

### 3.1 Clinical characteristics of mSTS patients

A total of 3078 eligible mSTS cases, diagnosed between 2016 and 2022 in the SEER database, were included in our study according to the inclusion and exclusion criteria. All patients were confirmed to have distant metastasis at diagnosis. In the entire cohort, 1594 patients (51.8%) were female and 1484 patients (48.2%) were male. 1842 (59.8%), 2195 (71.3%), and 1606 (52.2%) of the patients were younger (≤ 65 years), white, and married, respectively. In all patients, a substantial percentage of high-grade malignant tumors was observed: 224 cases (7.3%) were categorized as grade III, and 884 cases (28.7%) fell within the grade III-IV. Concerning treatment, 1378 (44.8%) patients underwent surgical intervention, 1858 (60.4%) received chemotherapy, and 829 (26.9%) underwent radiotherapy.

Then records of all subjects were randomly allocated into training (n = 1846) and validation (n = 1232) cohorts. The majority in both sets were younger (≤ 65 years), white, and married. The most common pathological type was osteosarcoma, and leiomyosarcoma. Lung was the most common metastatic site, followed by liver, bone, and brain. In both sets, most patients treated with chemotherapy or surgery. No significant disparities in demographic details, tumor types, or treatment modalities were observed between the training and validation groups. **Table 1** summarized the demographic and clinicopathological features of the patients in the two cohorts.

**Table 1:** Characteristics of patients with mSTS.

Characteristics	Total N (%)	Training cohort N (%)	Validation cohort N (%)	P-value
	(N=3078)	(N=1846)	(N=1232)	
Age				0.499
**≤ 65**	1842 (59.8%)	1089 (59.0%)	753 (61.1%)	
**>65**	1236 (40.2%)	757 (41.0%)	479 (38.9%)	
Sex				
Female	1594 (51.8%)	963 (52.2%)	631 (51.2%)	0.875
Male	1484 (48.2%)	883 (47.8%)	601 (48.8%)	
Race				
Black people	430 (14.0%)	262 (14.2%)	168 (13.6%)	0.994
White people	2195 (71.3%)	1311 (71.0%)	884 (71.8%)	
Other people	453 (14.7%)	273 (14.8%)	180 (14.6%)	
Marital status				1.000
Married	1606 (52.2%)	963 (52.2%)	643(52.2%)	
Unmarried	1472 (47.8%)	883 (47.8%)	589 (47.8%)	
Histologic subtype				0.947
Leiomyosarcoma	662 (21.5%)	388 (21.0%)	274 (22.2%)	
Liposarcoma	169 (5.5%)	106 (5.7%)	63 (5.1%)	
Myxofibrosarcoma	61 (2.0%)	42 (2.3%)	19 (1.5%)	
Osteosarcoma	850 (27.6%)	511 (27.7%)	339 (27.5%)	
Rhabdomyosarcoma	98 (3.2%)	57 (3.1%)	41 (3.3%)	
Synovial sarcoma	44 (1.4%)	31 (1.7%)	13 (1.1%)	
Other	1194 (38.8%)	711 (38.5%)	483 (39.2%)	
Pathological grade				0.938
I-II	224 (7.3%)	128 (6.9%)	96 (7.8%)	
III-IV	884 (28.7%)	532 (28.8%)	352 (28.6%)	
Unknown	1970 (64.0%)	1186 (64.2%)	784 (63.6%)	
Liver metastasis				0.995
No	2279 (74.0%)	1368 (74.1%)	911 (73.9%)	
Yes	799 (26.0%)	478 (25.9%)	321 (26.1%)	
Lung metastasis				0.669
No	1754 (57.0%)	1064 (57.6%)	690 (56.0%)	
Yes	1324 (43.0%)	782 (42.4%)	542 (44.0%)	
Brain metastasis				0.789
No	2998 (97.4%)	1801 (97.6%)	1197 (97.2%)	
Yes	80 (2.6%)	45 (2.4%)	35 (2.8%)	
Bone metastasis				0.784
No	2591 (84.2%)	1547 (83.8%)	1044 (84.7%)	
Yes	487 (15.8%)	299 (16.2%)	188 (15.3%)	
Metastasis number				0.583
0	987 (32.1%)	611 (33.1%)	376 (30.5%)	
1	1611 (52.3%)	944 (51.1%)	667 (54.1%)	
2	368 (12.0%)	217 (11.8%)	151 (12.3%)	
**≥** 3	112 (3.6%)	74 (4.0%)	38 (3.1%)	
Chemotherapy				
Not done	1220 (39.6%)	733 (39.7%)	487 (39.5%)	0.995
Done	1858 (60.4%)	1113 (60.3%)	745 (60.5%)	
Radiotherapy				
Not done	2249 (73.1%)	1352 (73.2%)	897 (72.8%)	0.966
Done	829 (26.9%)	494 (26.8%)	335 (27.2%)	
Surgery				
Not done	1700 (55.2%)	1023 (55.4%)	677 (55.0%)	0.968
Done	1378 (44.8%)	823 (44.6%)	555 (45.0%)	

### 3.2 Independent prognostic factors for mSTS patients

In the training cohort, the univariate Cox analysis demonstrated that age, sex, race, marital status, pathological grade, histologic subtype, liver metastasis, lung metastasis, brain metastasis, bone metastasis, metastasis number, surgery, radiotherapy, and chemotherapy were identified as statistically significant prognostic factors (**Table 2**). Among them, pathological grade (C-index = 0.631), chemotherapy (C-index = 0.619), surgery (C-index = 0.595), and age (C-index = 0.579) had higher discrimination ability in predicting OS than the other factors. Subsequently, multivariate Cox regression analysis was conducted using these statistically significant factors (**Table 3**). Finally, seven variables, namely age, race, marital status, pathological grade, histologic subtype, surgery, and chemotherapy, were identified as independent prognostic factors for OS, and used to construct the OS nomogram.

**Table 2:** Univariate Cox analysis of overall survival in mSTS (training cohort).

Variable	Reference	Characteristics	HR	Overall survival		
				95% CI of HR	P-value	C-index
**Age**	≤ 65	>65	1.69	1.48–1.94	<0.001	0.579
**Sex**	Female	Male	0.97	0.85–1.11	0.65	0.505
**Race**	Black people	White people	0.96	0.79–1.17	0.69	0.519
		Other people	0.73	0.57–0.95	0.02	
**Marital status**	Married	Unmarried	1.25	1. 11–1.43	0.001	0.534
**Histologic subtype**	Leiomyosarcoma	Liposarcoma	1.23	0.91–1.67	0.1709	0.631
		Myxofibrosarcoma	0.73	0.42–1.28	0.274	
		Osteosarcoma	1.46	1.22–1.75	<0.001	
		Rhabdomyosarcoma	1.59	1.11–2.27	0.011	
		Synovial sarcoma	1.20	0.64–2.26	0.577	
		Other	0.53	0.44–0.63	<0.001	
**Pathological grade**	I-II	III-IV	1.71	1.32–2.21	<0.001	0.544
		Unknown	1.24	0.97–1.60	0.089	
**Liver Metastatic**	No	Yes	1.00	0.86–1.16	0.97	0.500
**Lung Metastatic**	No	Yes	1.75	1.53–2.01	<0.001	0.570
**Brain Metastatic**	No	Yes	2.14	1.47–3.11	<0.001	0.510
**Bone Metastatic**	No	Yes	1.61	1.35–1.91	<0.001	0.527
**Metastasis numbers**	0	1	1.63	1.39–1.92	<0.001	0.576
	0	2	2.44	1.96–3.05	<0.001	
	0	≥3	2.89	1.95–4.11	<0.001	
**Surgery**	Not done	Done	0.58	0.50- 0.66	<0.001	0.595
**Radiotherapy**	Not done	Done	1.04	0.90–1.21	0.572	0.488
**Chemotherapy**	Not done	Done	0.55	0.48–0.63	<0.001	0.619

**Table 3:** Multivariate Cox analysis of overall survival in mSTS (training cohort).

Variable	Reference	Characteristics	HR	Overall survival	
				95% CI of HR	P-value
**Age**	≤ 65	>65	1.28	1.10–1.49	0.001
**Race**	Black people	White people	0.83	0.68–1.01	0.068
		Other people	0.71	0.54–0.92	0.009
**Marital status**	Married	Unmarried	1.33	1.16–1.52	<0.001
**Histologic subtype**	Leiomyosarcoma	Liposarcoma	1.27	0.93–1.73	0.133
		Myxofibrosarcoma	0.64	0.36–1.13	0.127
		Osteosarcoma	1.25	1.04–1.51	0.0163
		Rhabdomyosarcoma	1.85	1.28–2.66	0.001
		Synovial sarcoma	0.86	0.45–1.63	0.642
		Other	0.59	0.48–0.71	<0.001
**Pathological grade**	I-II	III-IV	1.87	1.43–2.45	<0.001
		Unknown	1.23	0.95–1.60	0.116
**Liver Metastatic**	No	Yes	1.53	0.44–5.36	0.505
**Lung Metastatic**	No	Yes	1.82	0.52–6.35	0.349
**Brain Metastatic**	No	Yes	2.03	0.61–6.74	0.245
**Bone Metastatic**	No	Yes	1.90	0.54–6.66	0.318
**Metastasis numbers**	0	1	0.87	0.25–3.05	0.829
	0	2	0.66	0.05–7.89	0.739
	0	≥3	0.51	0.01–23.96	0.733
**Surgery**	Not done	Done	0.55	0.47–0.64	<0.001
**Chemotherapy**	Not done	Done	0.58	0.50–0.67	<0.001

### 3.3 Nomogram construction

**Figure 1:**
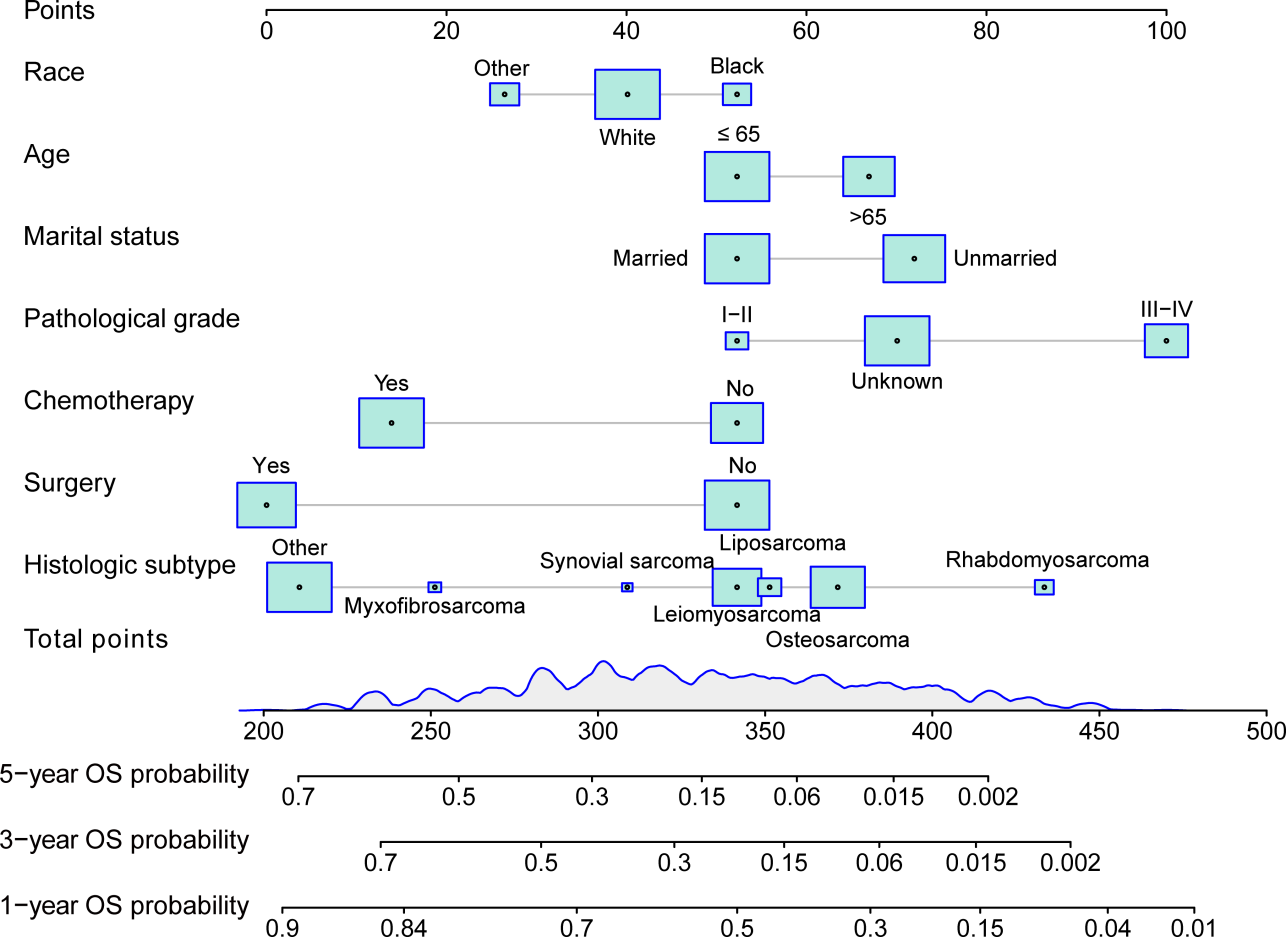
Nomogram for predicting 1-, 3-, and 5-year overall survival(OS) of patients with mSTS. The nomogram incorporated several key variables, including age, race, marital status, pathological grade, histologic subtype, surgery, and chemotherapy. These predictors were quantified as “point” based on patient-specific factors and then the sum of the “point” corresponded to the “total point” below, which corresponded to the 1-, -, 5-year OS. This total point score could be used to predict the patient’s overall survival probabilities at 3, 5, and 10 years, based on the nomogram’s predictive model. The higher the total point score, the lower the expected overall survival probability, allowing for a more personalized estimation of the patient’s prognosis.

A nomogram was devised to predict the 1-year, 3-year, and 5-year OS rates based on the significantly positive prognostic factors derived from multivariate Cox regression analysis, utilizing R software (**Figure 1**). Each prognostic factor was assigned a corresponding score on the upper scale, ranging from 0 to 100 points. The cumulative scores were then computed to determine the total points for variables, projected vertically downwards to ascertain the 1-, 3-, and 5-year OS rates. The prediction model highlighted pathological grade III-IV as the most critical factor influencing OS rate, followed by histologic subtype rhabdomyosarcoma, unmarried status, aged over 65 years, absence of surgery, absence of chemotherapy, and black race.

### 3.4 Nomogram performance and validation

**Figure 2:**
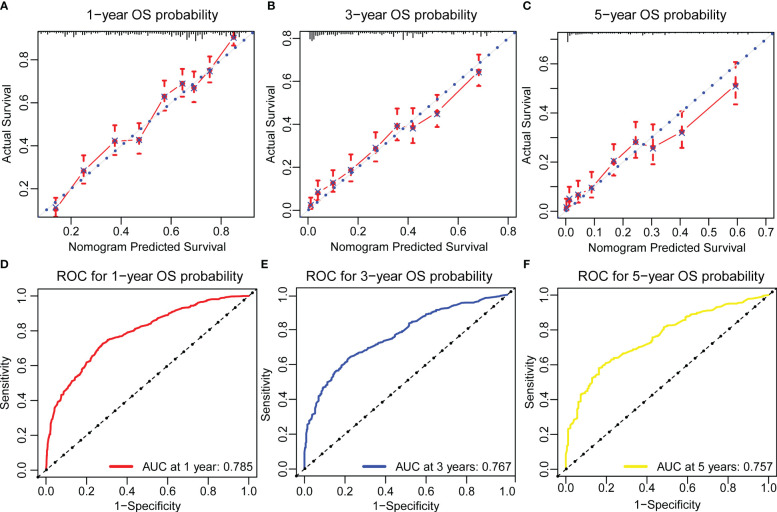
Calibration plots and ROC curves for predicting overall survival at 1- and 3- and 5-year in the training cohort. **(A–C)** The calibration plots for predicting patient survival at 1-, 3-,and 5-year in the training cohort. The predicted survival probabilities generated by the nomogram with the observed survival outcomes were compared. A perfect calibration was indicated when the plotted line aligns closely with the diagonal line. **(D–F)** ROC curves of the Nomogram in prediction of prognosis at 1-, 3-, and 5-year in the training cohort. The area under each ROC curve(AUC) quantified the nomogram’s discriminative power, where a higher AUC indicated better performance in distinguishing between survival and non-survival outcomes.

**Figure 3:**
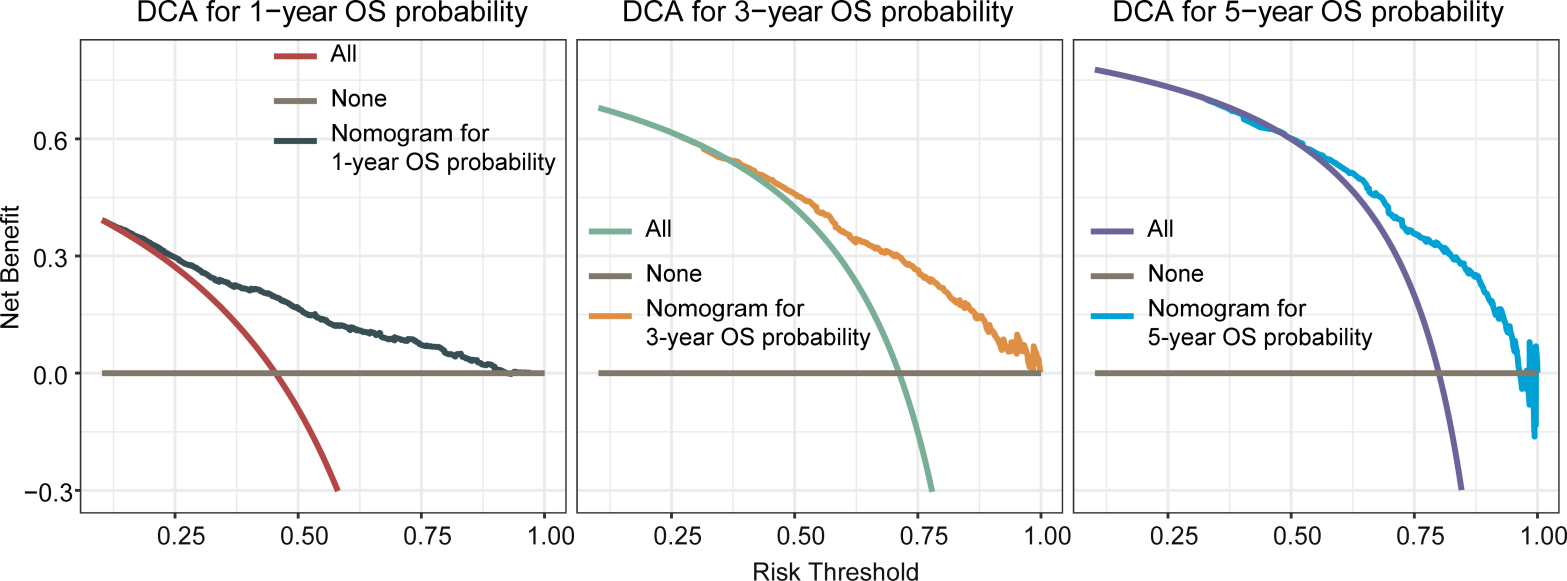
Decision curve analysis(DCA) of the nomogram for predicting overall survival at 1-, 3-, and 5-year in the training cohort. The x-axis represented the percentage of threshold probability, whereas the y-axis represented the net benefit, calculated by adding the true positives and subtracting the false positives. It allowed for evaluation of the potential of nomogram to improve decision-making compared to treating all or no patients. A higher net benefit at specific thresholds indicates the nomogram’s usefulness in guiding treatment decisions.

Based on the model we developed, we evaluated it performance in the training cohort. The c-index of the prognostic nomogram for predicting OS rates was 0.722 (95% CI: 0.708–0.736). The calibration plot of the OS nomogram demonstrated a high level of consistency between the predicted and actual OS (**Figures 2A–C** **).** ROC curves for 1-, 3-, and 5-year OS rates were generated, demonstrating significantly enhanced prediction accuracy. The AUC values of the nomogram were 0.785, 0.767, and 0.757 for the 1-, 3-, and 5-year OS rates, respectively (**Figures 2D–F**). Furthermore, the DCA indicated that the nomogram has great clinical utility in predicting 1-, 3-, and 5-year OS rates (**Figure 3**).

**Figure 4:**
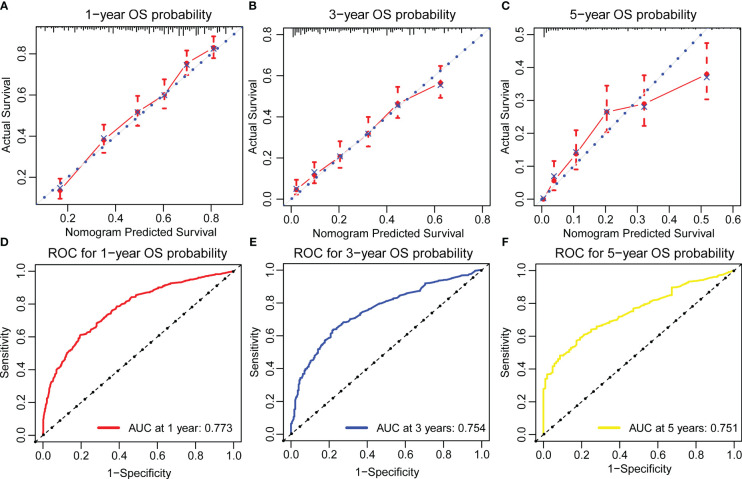
Calibration plots and ROC curves for predicting overall survival at 1-, 3-, and 5-year in the validation cohort. **(A–C)** The calibration plots for predicting patient survival at 1-,3-, and 5-year in the validation cohort. **(D–F)** ROC curves of the Nomogram in prediction of prognosis at 1-, 3-, and 5-year in the validation cohort.

**Figure 5:**
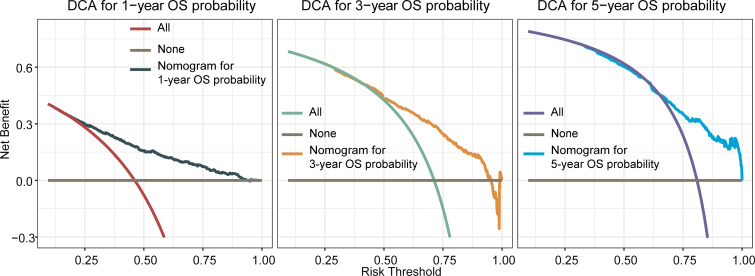
Decision curve analysis of the nomogram for predicting overall survival at 1-, 3-, and 5-year in the validation cohort. The x-axis represents the percentage of threshold probability, whereas the y-axis represents the net benefit, calculated by adding the true positives and subtracting the false positives.

The OS nomogram validation results in the validation cohort were as follows: the C-index value was 0.716 (95% CI: 0.698–0.734) and the calibration chart of the OS nomogram revealed high consistency between the predicted and actual data (**Figures 4A–C**). The AUC value of the ROC curve for 1-, 3-, and 5-year OS rates was 0.773, 0.754, and 0.751, respectively (**Figures 4D–F**). Cross-validation also revealed good accuracy and stability of the models and DCA demonstrated that the nomogram exhibited significant clinical utility for predicting 1-, 3-, and 5-year OS rates (**Figure 5**).

**Figure 6:**
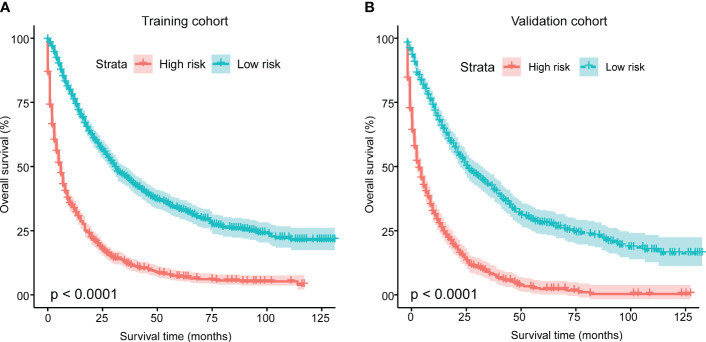
Kaplan-Meier analysis of overall survival for patients stratified by the risk stratification system in the training cohort **(A)** and validation cohort **(B)**. This system was based on the cumulative scores assigned to each patient with mSTS. Using the median total score as the cut-off point, patients were divided into two risk groups: a low-risk group and a high-risk group. The Kaplan-Meier curves depicted the differences in survival probabilities between two groups.

Furthermore, for the development of a risk stratification system utilizing this nomogram, we computed the cumulative score for each mSTS patient in the training cohort. Subsequently, we categorized individuals into two subgroups based on the median of total scores, denoting the low-risk group and, the high-risk group. Each delineated risk group exhibited a distinct prognosis, and the OS between the two subgroups was effectively delineated by the risk stratification system (**Figure 6**).

## 4 Discussion

Globally, the incidence of mSTS is on the rise. While many cases are identified early enough for successful surgical removal with a 5-year overall survival rate ranging from 60% to 80% (30). However, despite surgical intervention, approximately half of the patients experience local recurrence or distant metastasis, rendering surgery ineffective. The clinical, laboratory, and radiographic manifestations of STS collectively influence the natural course of the disease, which can vary from several months to several years based on its characteristics (31). Although the incidence rate of STS is low, at 0.029‰, the histological subtypes are complex (over 70 types), and significant differences exist in the molecular and biological characteristic among these subtypes. Among the different subtypes of STS, the angiogenic and tumor immune microenvironment is most prominent in the prevalent subtype (32). Prior to the advent of targeted and immunological therapies, mSTS posed significant treatment challenges with a grim prognosis. ICIs have now become a standard component of the treatment arsenal, including monoclonal antibodies targeting PD-1 and CTLA-4. The development of ICI, whether used alone or in combination with other agents, has yielded noteworthy results (12, 13). Our investigation was instigated by promising outcomes observed in clinical trials, particularly those involving ICIs. This study represented the largest cohort of patients with mSTS to the lung and liver treated with ICIs, revealing a significant improvement in survival.

Variation in metastatic sites concerning treatment modalities can inform clinical decision-making through relevant data sources. While liver metastasis has been demonstrated to limit ICIs efficacy in patients and experimental models, the amalgamation of radiation targeted at the liver and ICIs has demonstrated the augmentation of anti-tumor immunity in cancer patients (33, 34). The optimal metastatic sites for radiation targeting to maximize immune-stimulatory effects in conjunction with ICIs remain unclear. Jiali et al. identified liver metastases as correlated with worse OS in melanoma patients, irrespective of tumor load, metastatic sites, age, gender, and therapy. Additionally, they confirmed that individuals with melanoma solely having liver involvement derived lesser benefit from ICIs compared to those with lung involvement (35). Furthermore, a study on gastric cancer suggests that individuals with lung or liver metastasis have a higher likelihood of developing bone metastasis than those without lung or liver involvement (36). The multivariable Cox analysis substantiated that OS in mSTS with lung metastasis surpassed that of other metastatic groups (brain, bone, liver) in the era of immune ICIs. To our knowledge, this represented the most extensive population-based study to date investigating the survival advantages of ICIs in mSTS across various metastatic sites.

In the realm of treatment, employing chemotherapy and surgery as monotherapies in mSTS results in enhanced survival outcomes compared those patients did not undergo. Conversely, there is no observed alteration in survival rates associated with radiotherapy, possibly attributed to the noteworthy influence of recently identified targeted treatments. Surgery is a cornerstone in the treatment of STS. Direct removal of the primary tumor through surgical excision not only alleviates patient symptoms but also provides essential histological diagnosis. It is noteworthy that almost all patients (96.38%) in our study underwent surgery, which might account for this outcome. For early-stage STS, surgery is often the preferred primary treatment. However, for advanced or locally advanced cases, previous studies have proposed that surgery may be combined with radiation or chemotherapy to optimize therapeutic outcomes compared to surgery alone, such as mastectomy (37, 38). Chemotherapy plays a crucial role in STS treatment, employing pharmaceutical intervention to disrupt cancer cell growth and division. For highly malignant mSTS, chemotherapy is frequently utilized as part of a comprehensive treatment plan, either before or after surgery (39). Sensitivity to chemotherapy varies based on the subtype and molecular characteristics of STS, necessitating personalized treatment approaches. Our findings indicated that chemotherapy did confer a prognostic benefit for mSTS patients across all molecular subtypes. However, the C-index of chemotherapy was lower than that of pathological grade, highlighting that the pathological grade, dictated the effectiveness of chemotherapy in improving prognosis for mSTS patients (40). Radiation therapy plays a pivotal role in STS treatment, serving as both neoadjuvant and adjuvant therapy. Neoadjuvant radiation aims to reduce tumor volume before surgery, enhancing the chances of successful surgical intervention (41). Additionally, adjuvant radiation post-surgery helps minimize the risk of local recurrence. Recent advancements in radiation techniques, such as 3D conformal radiation and intensity-modulated radiation, have significantly improved precision and therapeutic efficacy. Furthermore, our analysis revealed that mSTS patients failed to derive benefits from radiotherapy in terms of OS. It is important to note that this should not undermine the significance of radiotherapy as its primary role is to reduce local recurrence rates following mastectomy, chemotherapy, and axillary lymph node dissection (42, 43). Despite radiotherapy not emerging as independent prognostic factors in our study, they continued to be pivotal components of mSTS treatment. Notably, no prior studies have investigated the factors influencing the effectiveness of radiotherapy specifically for mSTS patients in the ICIs rea, prompting us to conduct a more detailed stratified analysis for radiotherapy. In summary, our findings underscored the critical role of chemotherapy and surgery for mSTS patients, significantly contributing to their OS benefits.

Various scoring systems serve predictive purposes. Despite their simplified clinic utilization, each patient undergoes a stratified population risk assessment. Nomograms prove valuable in assessing patient survival outcomes, employing statistical modeling and risk quantification to navigate the complexity of balancing multiple factors. Their systematic approach mitigates the impact of individual physicians’ biases or anomalous clinical factors, surpassing typical stage score methods in accuracy (17–19). Nomograms excel in situations where the potential benefits of additional therapy remain uncertain (44). They also prove beneficial for personalized risk assessment, aiding clinicians in clinical care management when specific guidelines are absent. Given the substantial impact of metastasis on prognosis in the era of ICIs, we aimed to construct a comprehensive and practical nomogram for predicting the survival probability of mSTS patients. Several nomograms for mSTS have been formulated to predict recurrence and survival. In 2023, Callegaro et al. focused on the role of systemic inflammatory indices, particularly the lymphocyte/monocyte ratio and trabectedin, in second-line STS patients. This study highlighted the growing recognition of inflammatory indices as potential prognostic parameters, offering new avenues for enhancing patient care and outcomes (21). In 2024, the study by Callegaro et al. introduced new prognostic nomograms for patients with primary retroperitoneal STS, to update the current RPS prognostic nomograms considering the improvement in patient prognosis and the case volume effect (20). Our nomograms demonstrated commendable calibration and discrimination, as indicated by the C-index, calibration curves, ROC, and DCA. These straightforward nomogram models serve as therapeutic aids, enhancing patient counseling and facilitating treatment individualization.

The correlation between marital status and survival has been investigated across various tumors, including breast cancer, rectal cancer, and metastatic renal cell carcinoma (45–47). Our analysis revealed marital status as an independent prognostic factor in the univariate assessment, indicating that married patients exhibited a reduced mortality risk. Upon adjusting for demographic and clinical factors, married individuals demonstrated a lower mortality risk compared to their unmarried counterparts. A previous study focusing on renal cell carcinoma patients by Zhang et al. found that single individuals faced an elevated risk of tumor-related death (48). In studies involving STS, marriage conferred greater protection against poorer prognosis (28, 49). A comprehensive analysis of over a million patients diagnosed with various diseases indicated that unmarried individuals had a heightened likelihood of metastatic cancer and cancer-related mortality (50). Consistent with these findings, our results underscored the association between marriage and enhanced survival. Two potential explanations may account for the longer lifespan observed in married patients. Firstly, married individuals likely underwent regular physical examinations overseen by their spouses, facilitating early disease detection. Additionally, spouses may contribute more substantial economic support for subsequent treatments. Secondly, cancer patients are over four times more prone to experiencing psychological disorders (51). Following a cancer diagnosis, married individuals exhibited reduced despair and psychological distress, attributable to the encouragement and support from their spouses (46, 52, 53).

However, our research has certain limitations. Firstly, the study utilized the SEER database, primarily comprising data from American patients, leading to a predominant representation of white people and black people, with a comparatively smaller proportion of Asians, thereby introducing bias into our model’s predictions for Asian populations. Moreover, the study spanned from 1998 to 2016, during which there were advancements in diagnostic imaging and treatment methods, potentially influencing the prediction outcomes. Thirdly, our sample screening process involved excluding cases that did not meet inclusion criteria or had missing data, introducing a degree of selection bias. Lastly, despite the population-based nature of the SEER database and internal validation of the nomogram, external data were not utilized to verify the accuracy of the predictive nomogram, which is essential for confirming the model’s reliability. In addition, the nomogram failed to incorporate all possible factors that could impact patient survival outcomes, such as laboratory tests and systemic inflammatory indices (21). This was due to the lack of comprehensive data on these variables in the available SEER database. Furthermore, more detailed information on individual heterogeneity, such as specific molecular markers and genetic profiling, was not included. These factors could have provided a more nuanced understanding of the prognosis and potentially enhanced model’s performance. Future studies may benefit from considering these additional variables to enhance the depth and reliability of prognostic models for patients with mSTS.

## 5 Conclusion

A nomogram was formulated for prognosticating 1-, 3-, and 5-year OS rates in patients with mSTS undergoing surgical intervention. Our findings indicated the potential advantages of incorporating chemotherapy and surgery in the management of these patients. These nomogram models offered a practical and informative resource for clinicians and patients, facilitating the assessment of the anticipated advantages associated with chemotherapy and surgery and offering valuable guidance for the treatment of mSTS.

## Data availability statement

The original contributions presented in the study are included in the article/supplementary material. Further inquiries can be directed to the corresponding authors.

## Author contributions

DH: Writing – original draft. BL: Writing – original draft. JX: Writing – original draft. YH: Writing – original draft. XC: Writing – review & editing. RW: Writing – review & editing.
